# Characterization of environmental factors in children and adolescents with cerebral palsy in Minas Gerais: Participa Minas

**DOI:** 10.1590/1984-0462/2024/42/2023043

**Published:** 2024-02-12

**Authors:** Maria Luíza de Faria Alves, Deisiane Oliveira Souto, Angélica Cristina Sousa Fonseca Romeros, Elton Duarte Dantas Magalhães, Larissa Gabrielle Mendes, Kênnea Martins Almeida Ayupe, Paula Silva de Carvalho Chagas, Ana Carolina de Campos, Rafaela Silva Moreira, Aline Martins de Toledo, Ana Cristina Resende Camargos, Egmar Longo, Hércules Ribeiro Leite, Rosane Luzia de Souza Morais

**Affiliations:** a Universidade Federal dos Vales do Jequitinhonha e Mucuri, Diamantina, MG, Brazil.; b Universidade Federal de Minas Gerais, Belo Horizonte, MG, Brazil.; c Universidade de Brasília, Brasília, DF, Brazil.; d Universidade Federal de Juiz de Fora, Juiz de Fora, MG, Brazil.; e Universidade Federal de São Carlos, São Carlos, SP, Brazil.; f Universidade Federal de Santa Catarina, Araranguá, SC, Brazil.; g Universidade Federal do Rio Grande do Norte, Santa Cruz, RN, Brazil.

**Keywords:** Cerebral palsy, Children, Functioning, Environment, Paralisia cerebral, Crianças, Funcionalidade, Ambiente

## Abstract

**Objective:**

To characterize the environmental factors of children and adolescents with Cerebral Palsy (CP) in the state of Minas Gerais (MG), Brazil.

**Methods:**

This is a cross-sectional study involving 164 caregivers of children/adolescents with CP, aged 1-14 years. The Gross Motor Function Classification System (GMFCS) and the Manual Ability Classification System (MACS) were used to classify the participants’ functioning, and environmental factors were evaluated by an on-line questionnaire that examined products and technologies, physical environment, services, and systems. A descriptive analysis was performed using percentage and frequency.

**Results:**

Most participants had bilateral CP (66.9%) and 45% of them were spastic. Levels II and V of the GMFCS and MACS were the most frequent. About half (49.4%) used anticonvulsants, 27.4% underwent *botulinum* toxin application, and 29% went through orthopedic surgery in the lower limbs. Among the participants, 71.3% used orthoses in the lower limbs, and 51.8% used the public health care system. Most had access to physiotherapy (91.5%), but found difficulties to access interventions with other professionals, such as psychologists (28%) and nutritionists (37.8%). The school was the most frequently adapted environment (78%), and had the highest level of structural adaptation (42.7%).

**Conclusions:**

The results of this study suggest that the barriers to access health services and barriers to the physical environment may impact participation and social inclusion.

## RESUMO

**Objetivo:**

Caracterizar os fatores ambientais de crianças e adolescentes com paralisia cerebral (PC) no estado de Minas Gerais (MG), Brasil.

**Métodos:**

Trata-se de um estudo transversal envolvendo 164 cuidadores de crianças/adolescentes com PC, na faixa etária de um a 14 anos. O Sistema de Classificação da Função Motora Grossa (GMFCS) e o Sistema de Classificação da Habilidade Manual (MACS) foram utilizados para classificar a funcionalidade dos participantes e os fatores ambientais foram avaliados por um questionário *on-line* que abordou produtos e tecnologias, ambiente físico, serviços e sistemas. Análises descritivas foram realizadas por meio de porcentagem e frequência.

**Resultados:**

A maioria dos participantes tinha PC bilateral (66,9%) e 45% deles eram espásticos. Os níveis II e V do GMFCS e MACS foram os mais frequentes. Cerca de metade (49,4%) fazia uso de anticonvulsivantes, 27,4% realizaram aplicação de toxina botulínica e 29% cirurgia ortopédica em membros inferiores. Utilizavam órteses em membros inferiores 71,3% e eram usuários do sistema público de saúde 51,8%. A maioria tinha acesso à fisioterapia (91,5%), mas dificuldade de acesso a intervenções com outros profissionais, como psicólogos (28%) e nutricionistas (37,8%). A escola foi o ambiente mais frequentado (78%) e também mais adaptado estruturalmente (42,7%).

**Conclusões:**

Os resultados deste estudo sugerem que barreiras de acesso aos serviços de saúde e barreiras no ambiente físico podem impactar a participação e inclusão social.

## INTRODUCTION

Cerebral Palsy (CP) is a group of permanent disorders of movement and posture, due to non-progressive changes that occur in the brain during the early stages of development.^1^ The health status of children with CP may be compromised in all domains of functioning described by the International Classification of Functioning, Disability and Health - ICF,^1^ and the environment in which the child is included is an important determinant of the health condition.^2^ ^,^ ^3^ ^,^ ^4^ The literature has shown that the environmental factors, such as support and relationships, attitudes and services, systems and policies, are highly relevant for the functioning of children with CP.^4^

Child development is the result of the interaction between genetic inheritance and environmental factors. According to the ICF, environmental factors can be defined as the physical, social and attitudinal environment in which people live and conduct their lives.^1^ They include products and technologies, natural environment and environmental changes, supports and relationships, services, social systems and policies.^1^

Studies conducted in high-income countries highlight the importance of approaches aimed at identifying aspects of the environment and activity that support or hinder the participation of children and adolescents with CP in different contexts.^5^ ^,^ ^6^ ^,^ ^7^ Moreover, there is evidence that environmental factors play a significant role in participation.^6^ Despite the importance of environmental factors for the functioning of children and adolescents with CP, there is a paucity of studies evaluating environmental factors in low- and middle-income countries.^8^ There is early evidence that children and adolescents with CP in low- and middle-income countries have limited access to rehabilitation and education services.^9^ Brazil is a country with diverse socioeconomic and cultural conditions and faces many challenges, such as access to public services and evidence-based rehabilitation.

In addition to the unavailability of studies investigating environmental factors in Brazil, the prevalence of CP is also uncertain, since there is no data available from CP records.^10^ The environmental factors related to Brazilian children and adolescents with CP remain unknown in the literature. Thus, the present study aimed to characterize the environmental factors regarding children and adolescents with CP in the State of Minas Gerais (MG), Brazil. According to the Brazilian Institute of Geography and Statistics (IBGE) the human development index (HDI) in Brazil is 0.759, and the State of Minas Gerais is a reflection of the reality of Brazil considering the heterogeneity of its socioeconomic indicators.^11^ On the one hand, there are meso regions with a high (HDI), such as the Center-South and *Triângulo Mineiro*, and others with less economic dynamism, such as the East and North zones, all within the State of Minas Gerais, which offer lower living conditions for the population, resulting in an average state HDI of 0.731.^11^ The characterization of environmental factors is important to plan interventions and encourage the development of public policies that favor the functioning of these children.

## METHOD

This is a descriptive, cross-sectional study focused on the State of MG (PartiCipa Minas), which is part of a longitudinal multicenter project called “Activity Curves and Participation Trajectories for Children and Adolescents with Cerebral Palsy - PartiCipa Brasil.”^10^ The present study was approved by the Ethics and Research Committee of the Federal University of Juiz de Fora (UFJF), under opinion number 4.418.388.

The sample was obtained by convenience, recruited from direct contacts made available by the Federal Institutions of Higher Education in the State of Minas Gerais that assist children/adolescents with CP in their university hospitals. All individuals who responded to the contact between March 2021 and July 2022 were invited to participate in this study. Parents and caregivers of children/adolescents diagnosed with CP, aged between 1 and 14 years, of all clinical types and levels of the Gross Motor Function Classification Measure (GMFCS),^12^ were included in the study. The GMFCS family report version^12^ was used to characterize the participants’ gross motor activity levels. To characterize the manual ability of children with CP, the Manual Ability Classification System (MACS) was used.^13^

To collect the environmental factors, an on-line questionnaire (Google Forms) was developed based on the conceptual structure of part II of the ICF (Contextual Factors). The questionnaire was developed by a group of 11 researchers, nine professors from IFES in Brazil, and two from Centers of Excellence in Research in the United States and Canada. The variables collected by the questionnaire were: socioeconomic data, including the caregiver’s education, family income and access to the Social Security Benefit (SSB); medical interventions; assistive technology; transport, health, and education services; health services by specialty; and physical environments.

Data collection was conducted through a questionnaire to the main caregivers of the participants, between March 2021 and July 2022, remotely or in person, according to availability and scheduling, as it occurred during COVID-19 isolation period. Prior to the start of data collection, all participants signed the Consent Term and the objectives, benefits, and risks of participating in the study were clarified. During the remote application of the instruments, one of the researchers remained online to answer any questions from the participants. Six graduate students took part in the procedures, participants of the “PartiCipa Brasil” Project residing in the State of MG, previously trained to apply the instruments.

Data were organized in the statistical program Statistical Package for the Social Science (SPSS) version Windows 22.0^®^, and later submitted to descriptive analysis with measures of central tendency, dispersion, percentage, and frequency.

## RESULTS

A total of 164 parents and caregivers, along with their children and adolescents with CP, participated in the study. The results were predominantly obtained through forms applied remotely (94.5%).

**Figure 1.**
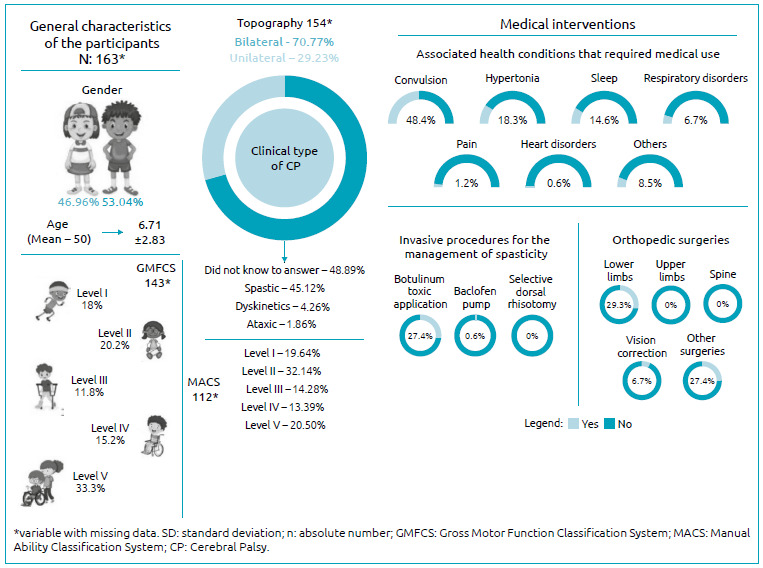
Descriptive characteristics of participants and medical interventions.

The personal characteristics of the children/adolescents are shown in Figure 1. The age ranged from 1 to 14 years, with most being male and having received the diagnosis before the first year of life. Almost half of the caregivers were unable to inform the participant’s clinical type of CP, and most of those who knew their child’s classification reported that the participants had spastic CP. Most participants had bilateral motor impairment. Regarding functional classifications, levels II and V of the GMFCS and MACS were the most predominant.

Approximately 64% of primary caregivers had at least completed high school. Family income data were collected according to the value of the minimum wage established in Brazil, and it was observed that about 41% received between 1 and 2 minimum wages, 32.4% had an income greater than two minimum wages, and 26.6% received up to one salary. Approximately 38.4% received the Social Security Benefit and, of these, 76.4% faced difficulties in receiving the benefit.

Figure 1 contains descriptive data about the medicalfn interventions performed by the participants of this study. As for the use of medication, almost half used anticonvulsants, and a small percentage used analgesics. Although most children/adolescents had the clinical spastic type, less than 1/3 had *botulinum* toxin type A application to manage spasticity; furthermore, other resources, such as selective dorsal rhizotomy, were not used. Orthopedic surgeries to correct musculoskeletal changes in the lower limbs were the most frequent. Other surgeries were also reported, such as appendectomy, tracheostomy, gastrostomy, inguinal hernia, implantation of ventriculoperitoneal shunt.

**Figure 2.**
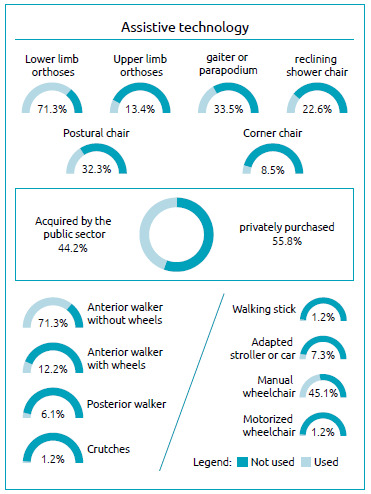
Assistive technologies.

The use of assistive technology by the participants of this study is represented in Figure 2. Most children used lower limb orthoses (71.3%), while other assistive technologies were little used. Only 13.4% of children/adolescents used orthoses for the upper limbs. Lower limb extensor splints (gaiters) or vertical stabilizers (parapodium) were used by 33.5% of the participants. In most cases, these technologies were acquired through their own financial resources.

Among the participants, 22.6% used the bathing chair, while 32.3% used the postural chair. A small percentage used a corner chair (8.5%), adapted strollers (7.3%), posterior walkers (6.1%) and anterior walkers with wheels (12.2%). Most of these technologies were acquired through their own financial resources. Only three participants used an anterior walker without wheels, two of them provided by the public health care system. Only four children benefited from the use of crutches and canes, two provided by the public health care system, and two acquired with their own financial resources. 45.1% used a manual wheelchair, of which 22.6% were provided by the public health care system, while only two children/adolescents used a motorized wheelchair, one provided by the public health care system.

Table 1 presents the transport, health and education services used by children/adolescents. Public transport was rarely reported (public bus=36%, municipal transport=5.5%, health service vehicle=1.8%), private car being the main means of transport for children/adolescents (52.4%). On the other hand, when it comes to health services, little more than half of the participants used the public health care system (50.8%). As for the educational service, most of the children/adolescents attended public schools (58.8%). However, almost a third of the children did not attend schools (22.0%), and a small number attended specialized public or private non-profit institutions (9.8%).

**Table 1.** Transport, health, and education services.

Characteristics	Frequency (%)
Transport (n=159)*	
Own car	86 (52.4)
Public bus	59 (36.0)
Municipal transport	9 (5.5)
Health service vehicle	3 (1.8)
Walk	2 (1.2)
Health service (n=162*)	
Public health care system	85 (50.8)
Supplementary health	77 (47.2)
Education (n=163*)	
Public school (regular)	94 (58.8)
Doesn’t attend	36 (22.0)
Private school (regular)	17 (10.4)
Special public school	16 (9.8)

*variable with missing data.

**Figure 3.**
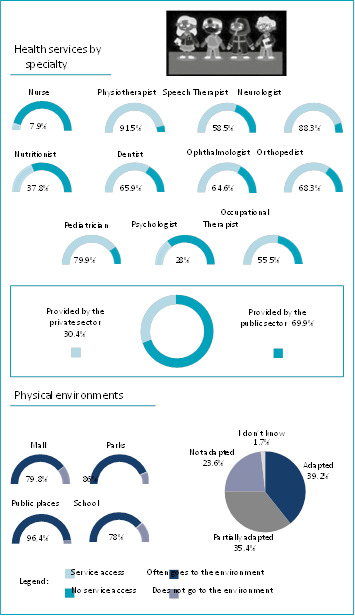
Health services and physical environment.

Detailed information on health services by specialty is shown in Figure 3. With regard to rehabilitation services, physiotherapy was the most used specialty (91.5%), followed by dentistry (65.9%), speech therapy (58.5%) and occupational therapy (55.5%). Other specialties, such as nutrition, psychology and nursing, were less frequent.

The public health care system provided almost all physiotherapy (64%) and occupational-therapy (35.4%) services. A little more than half of the psychology consultations (54.34%) and about 66% of the speech therapy consultations were also provided by the public health care system. In the case of dental care, around 42% were accessed through supplementary health services.

Of the medical specialties, most of the participants were assisted by neurology, pediatrics, orthopedics and ophthalmology. The consultation with neurologists, in 45.2%, was performed within the public health care system. However, just under half of the orthopedic, ophthalmic, and pediatric care was provided by the public health care system. These were mostly obtained through supplementary health services.

Figure 3 also shows the total percentages of adaptations in environments frequented by children and adolescents with CP. Schools were the least frequented physical environment and had the lowest environmental barriers (42.7%), with greater adaptations to the needs of children/adolescents with CP. On the other hand, parks were identified as the biggest environmental barriers (36%). On the other hand, public places appear with high percentages of partially adapted accessibility (39.6%).

## DISCUSSION

The present study characterized the environmental factors of children and adolescents with CP residing in the State of MG, Brazil.

The results of the present study point to anticonvulsants as the most used drugs by children/adolescents with CP. These drugs are used to control epilepsy in order to prevent seizures. The high prevalence of epilepsy in individuals with CP has been reported in previous studies.^14^ Bruck et al.^15^ showed that the prevalence of epilepsy in a sample of Brazilian children was 62%. In a characterization study of Northeastern children with CP, the authors also found epilepsy as the most common comorbidity.^16^ Although seizures can be controlled by pharmacotherapy, epilepsy remains one of the main causes of anxiety for the family of children and adolescents with CP.^15^ There is still evidence that the presence of seizures in the previous year was associated with a significant reduction in the quality of life^17^ and, in this perspective, the medication control of epilepsy can play the role of an environmental facilitator.

Our results indicate that spastic CP was the most frequent in the population studied, as in other studies in Brazilian regions. Even though most participants had spastic CP, reflecting the most common type of CP worldwide,^18^ almost half of caregivers were unable to inform the participant’s clinical type of CP. This result reflects a lack of knowledge translation and information communication provided by healthcare professionals in Brazil. Specifically, it highlights the issue of healthcare professionals not informing parents about the type of CP that their child or adolescent has. This lack of communication can lead to a gap in understanding and awareness among parents regarding their child’s condition, potentially hindering appropriate support and decision-making.

Early management of spasticity may favor the acquisition of gross motor function.^19^ The literature recommends the combination of physiotherapy, use of orthotics and assistive devices, and *botulinum* toxin injections for early management of spasticity.^19^ The use of these strategies can minimize or delay the need for surgical interventions. Nevertheless, in this study, orthopedic surgeries on the lower limbs (29%) were more frequent than *botulinum* toxin applications (27%). In children with bilateral CP, early tendon lengthening surgery to replace *botulinum* toxin resulted in a severe squat gait in more than 40% of the children studied.^19^ Thus, the use of technologies and application of *botulinum* toxin, both available through the Brazilian public healthcare system, must be considered the first choice in the management of spasticity, and can minimize the need for surgical procedures, which, in addition to being expensive, when performed early, can result in complications and worsen the physical conditions of children.

However, in some cases, the orthopedic surgeries can minimize impairments and activity limitations associated with the musculoskeletal system.^20^ ^,^ ^21^ In the present study, approximately 30% of the participants underwent corrective surgery on the lower limbs. Orthopedic procedures are designed to address different musculoskeletal complications of children with CP, including tendon stretches or transfers, rotational osteotomies, and joint stabilization procedures.^20^ All these procedures are provided by the public health care system. However, in practice, the approval of such procedures tends to be slow and bureaucratic, which can make it difficult to perform them in the appropriate timeframe, or even lead to a decline in functioning.^22^

In children with CP, assistive technologies can minimize difficulties and facilitate functioning in everyday activities.^23^ Lower limb orthoses were the devices most used by participants in the present study. On the other hand, other types of assistive technologies were little used. It was not possible to verify whether the limited use of some of these technologies was due to the fact that they were not necessary for the population studied or due to lack of access. The Brazilian public health service provides a table with all procedures, medications and orthoses, prostheses and special materials available for prescription by health professionals to assist Brazilian people with disabilities. However, this table is outdated, with new procedures and products not included and values not adjusted for approximately 14 years. In addition, the prescription products take a long time to be delivered by the public health system. This justifies the fact that most of the assistive technologies acquired by the participants of this study were purchased through their own financial resources. Some important assistive technologies commonly used by individuals with CP are not included in the subtype of public health care system funding, such as posterior walkers, powered wheelchairs (i.e., for children under 12 years old) and parapodium. Resources such as transfers, gait trainers and adapted strollers or cars are also not available on the public health care system. These devices have been identified as excellent ways to promote activity and participation.^24^ In this study, only 7.3% of children used adapted strollers or cars, and 1.2% used a motorized wheelchair for locomotion. These technologies are especially important for children classified at GMFCS level V. Considering that about 33% of the children studied were classified at this level, our data possibly indicates that most of these children did not have access to these technologies for locomotion. These findings reinforce the need to expand the supply of assistive technologies for mobility and locomotion by the public health care system, since the use of these technologies can assure greater autonomy for children and reduce the workload for caregivers.In summary, *botulinum* toxin, orthopedic surgery and assistive technology are environmental facilitators, as long as they are indicated considering the particularities of each case.

The results of the present study indicated that only 51.8% of participants with CP use the public health care system for health care, while approximately 47.2% used supplementary health. These results corroborate the hypothesis by Tostes et al.^25^ that people with CP generally receive the necessary treatment through private health plans. The National Health Policy for People with Disabilities ensures access to health through the public health care system for people who have some type of disability.^26^ Even after three decades of implementation of the public health care system, the population with disabilities still lack access to the public health system, which makes it difficult to plan health actions aimed at the needs of this population.^27^ A study conducted in southern Brazil aimed to identify the factors that make it difficult for children and adolescents with physical disability to access physiotherapy treatment in the public health system, and identified as main factors: the distance between the establishment and the residence of the users, little offer of services, waiting list and lack of financial resources.^28^

CP is a chronic condition that has no cure and, given its complexity, care will only be complete through the involvement of a multidisciplinary team. The multidisciplinary approach includes medical follow-up, assessment by psychologists, interventions with physiotherapists, speech-therapists, occupational-therapist, among others.^26^ A small percentage of children are assisted by other professionals, such as nutritionists and psychologists. These results point to the need to expand the network of specialized services offered by the public health care system, given that most participants use the network of private services, although most of them have a low socioeconomic level. Thus, a multidisciplinary and integrative approach to rehabilitation should be provided as an effective way to manage this health condition.^26^

In summary, although it is free, there are still barriers to access the public health care system and multidisciplinary team in Brazil.^28^

Several decrees have been drafted in Brazil and around the world to guarantee the school inclusion of children with disabilities. However, there is a lack of conditions to assure not only access to schools, but also the permanence and success of these students in regular classes, with specialized professionals prepared to meet their needs.^28^ Consistent with this statement, only 58.8% of the participants in the present study attended regular schools. In addition, a worrying finding is that about 22% of participants did not attend school. The low attendance may be linked to several factors, including the absence of adequate assistive technologies that can guarantee greater mobility in the school environment. Other factors that may be related are architectural and attitudinal barriers, which act as important obstacles to participation. Non-adapted environments have a major impact on these children’s activity and participation levels.^24^ Therefore, it is necessary to minimize architectural and attitudinal barriers, and the school space must be structured so as to offer assistive technology resources, services and strategies.^29^

Aspects of the physical environments visited by children with CP were also addressed in the present study. Approximately 42% of children pointed to school as being an environment with adapted accessibility. Thus, it is likely that the lack of adaptations in the school environment is one of the factors explaining why 1/4 of the children and adolescents in this study do not attend schools. The environment has been considered a potentially modifiable variable to increase the accessibility and participation of children/adolescents.^30^ ^,^ ^31^ There is evidence that children with CP and similar degrees of motor impairment have different levels of participation, which reinforces the potential effect of the environment.^32^ The identification of environmental barriers and facilitators are fundamental aspects to ensure the participation of children and adolescents with CP at home, at school and in the community.

This study has some limitations. The number of participants invited to participate and who did not respond to contact was not recorded. We can still mention the lack of knowledge about the number/prevalence of CP cases in the State of MG, which makes it difficult to generalize the results of this study to the entire group of children/adolescents with CP in the State or to other Brazilian States. In addition, all information was obtained from parents’ reports, including the classification systems, GMFCS and MACS, a necessary strategy considering that the procedures took place in the period of social isolation of the covid-19 pandemic, although both instruments have shown excellent agreement between caregivers and professionals. Despite this possible limitation, we believe that the parents are the best to report and state about their child/adolescent functioning and environmental factors. Finally, the environmental factors questionnaire did not allow us to identify whether assistive technologies not used by the population in this study were associated with unavailability or no need for use thereof.

In conclusion, the results presented by this study contributed to increase knowledge about the environment in which children/adolescents with CP live in a Brazilian State, and provided information that can help in the development of public policies that favor the health and well-being of this population.
